# The role of vitamin D deficiency and modifiable risk factors in patients with Crohn’s disease

**DOI:** 10.3389/fimmu.2025.1616924

**Published:** 2025-07-30

**Authors:** Xiaoyue Feng, Qin Yin, Ying Kang, Kang Jiang, Mengqing Xu, Fangyu Wang

**Affiliations:** ^1^ Division of Gastroenterology and Hepatology, Nanjing Jinling Hospital, Jinling School of Clinical Medicine, Nanjing Medical University, Nanjing, Jiangsu, China; ^2^ Orthopedics Department, Wuxi 9th People’s Hospital Affiliated to Soochow University, Wuxi, Jiangsu, China; ^3^ Division of Gastroenterology and Hepatology, Nanjing Jinling Hospital, Medical School of Nanjing University, Nanjing, Jiangsu, China

**Keywords:** vitamin D, Crohn’s disease, vedolizumab, age, LASSO regression analysis

## Abstract

**Backgrounds:**

Vitamin D insufficiency is usually seen in Crohn’s disease (CD). Our study aims to determine the risk factors for vitamin D insufficiency in CD patients.

**Methods:**

Between May 2021 and December 2023, we enrolled 102 CD patients and 100 healthy people in our hospital who were eligible for the study. Changes in vitamin D levels were also analyzed. CD patients were divided into active and clinical remission, and further changes in micronutrient and vitamin D levels were analyzed. Lastly, risk factor analysis was conducted using univariate, multivariate, and LASSO regression analysis models.

**Results:**

The average age of CD patients was 38.91 ± 3.31 years, whereas the average age of the healthy people was 38.64 ± 2.26 years. Vitamin D levels were significantly lower in CD patients than in healthy controls (19.62 ± 2.68 vs. 22.68 ± 4.61), especially for patients with active CD. In 11 patients treated with vedolizumab, compared to the pre-treatment Vedolizumab group, vitamin D levels improved more dramatically post-Vedolizumab therapy. According to univariate analysis, Age (OR: 0.95, 95% CI 0.26-1.33, p=0.01), sex (OR: 0.26, 95% CI 0.25-0.99, p=0.03), recent biologics (OR: 0.54, 95% CI 0.44-1.25, p=0.02), iron (OR: 0.89, 95% CI 0.72-1.62, p=0.02), and total 25-OH vitamin D (OR: 1.25, 95% CI 1.02-1.99, p=0.02) did significantly differ between patients with and without vitamin D deficiency. After controlling for several variables, multivariate analysis revealed that a lower odds ratio was linked to increasing age at diagnosis (OR: 0.12, 95% CI 0.03-0.85, p=0.02), sex (OR: 0.58, 95% CI 0.44-0.95, p=0.01), iron (OR: 0.44, 95% CI 0.11-0.62, p=0.01), and 25-OH vitamin D total (OR: 0.48, 95% CI 0.25-0.95, p=0.03). In addition, Age, time since illness onset, and 25-OH vitamin D were found to be helpful indicators for CD patients using LASSO regression.

**Conclusion:**

According to this study, vitamin D insufficiency was often linked to CD patients with active status and pre-treatment Vedolizumab. Furthermore, Age, time since illness onset, and 25-OH vitamin D were found to be significant risk factors for CD.

## Introduction

Abdominal discomfort, severe diarrhea, and exhaustion are clinical manifestations of Crohn’s disease (CD) (1), a chronic condition characterized by intestinal inflammation and gastrointestinal dysfunction (2). It has a significant impact on patients’ quality of life (3). Current treatment for CD includes biological agents, for example, vedolizumab is a gut-selective monoclonal anti-α4β7 integrin antibody treatment for Crohn’s disease (4). But there is still a risk of incomplete cure. Therefore, identifying potential risk factors for CD and alleviating symptoms while maintaining long-term remission are critical areas of research (5).

In the treatment of CD, inhibition of intestinal inflammation is key, and accurate assessment of disease severity is also essential (6, 7). Clinical symptoms, colonoscopy, gastroscopy, radiological imaging, and biopsy data from various gastrointestinal tract segments are usually used to evaluate disease activity (8). A full histological evaluation is performed to rule out chronic infectious bowel disease (9). However, endoscopic procedures for treatment and evaluation often carry risks such as high cost, complex surgery, and invasiveness (10, 11). Although different infections can alter their levels, the degree of CD has been assessed using fecal calprotectin and C-reactive protein (CRP) (12). Furthermore, CRP levels are influenced by hereditary variables, and fecal calprotectin is more effective when colonic mucosal inflammation is present (13). Finding trustworthy biomarkers for CD diagnosis and therapy is therefore still a top priority.

Vitamin D insufficiency, commonly defined as a blood 25(OH)D concentration below 50 nmol/L, is common in patients with Crohn’s disease (CD) (14). Vitamin D has a significant impact on intestinal homeostasis and lowers serum levels of inflammatory factors (15). Compared to patients with adequate vitamin D, those with vitamin D deficiency are more likely to require surgery (16–18). In addition, vitamin D supplementation tends to have benign benefits (16). In the absence of evidence-based recommendations for vitamin D optimization in IBD, treatment at levels of at least, but ideally, 75 ng/mL is safe and may improve IBD disease activity (19–21). Research has indicated that vitamin D levels significantly affect immunological regulation (22). In human PBMCs infected with Mycobacterium TB, 1,25D dose-dependently suppresses the production of the pro-inflammatory cytokines IL-6, tumor necrosis factor alpha, and IFNγ (23). The intrasecretory synthesis of 1,25D by CDs results in a tolerogenic phenotype, which is typified by decreased expression of major histocompatibility complex (MHC) class II, costimulatory markers including CD40, CD83, and CD86, and decreased production of IL-10, IL-12, and IL-23 (24).

In order to better understand the function of trace elements in these alterations, our study examined variations in vitamin D levels in individuals with active and remission CD. Lastly, to ascertain the function of risk variables in CD patients and to give predictors for risk factors and therapy, univariate, multivariate, and LASSO regression models were employed.

## Materials and methods

The institutional review board (IRB) and ethical committee of our hospital have authorized this single-center, controlled study (2021NZKY-001-01). After being informed, each patient signed an informed consent form. The CONSORT criteria state that these studies adhere to the Declaration of Helsinki.

### Patients

The study included both men and women aged 18 to 60 who were hospitalized for Crohn’s disease between May 2021 and December 2023. Measuring several parameters was the primary goal of the study. The healthy controls (n=100) were frequency-matched to CD patients by age (± 3 years) and sex (1:1 ratio).

Participants had to be between the ages of 18 and 60 and have a confirmed diagnosis of Crohn’s disease based on imaging, histology, endoscopy, and clinical symptoms (25). Data from gastrointestinal endoscopies and vitamin D availability. Individuals receiving vitamin D treatment were excluded, as were those with unclassified enterocolitis, pregnancy or lactation, alcohol or drug abuse, a history of mental or emotional disorders, or a history of severe or chronic cardiovascular, respiratory, urinary, endocrine, reproductive, skeletal, muscular, neurological, or other systemic disorders. Exclusion criteria for all participants (CD and controls) included: a) Vitamin D/calcium supplementation (>400 IU/day) within 3 months; b) Malabsorption syndromes (e.g., celiac disease, pancreatic insufficiency); c) Severe renal/liver impairment (eGFR <60 mL/min/1.73m² or ALT >3× ULN); d) Pregnancy or lactation.

Patients who are thinking about stopping vedolizumab within the next four months or who have been taking it for less than six months. The study’s controls were healthy volunteers who did not have Crohn’s disease and who satisfied the inclusion requirements. Patients with vitamin D insufficiency (25-OH-D <30 ng/mL) received cholecalciferol 800 IU/day throughout vedolizumab therapy, aligning with ECCO guidelines on micronutrient repletion in active IBD. Supplementation commenced ≥4 weeks pre-treatment and was maintained during the 14-week observation period. Blood samples for vitamin D (25-OH-D) were drawn between 8–10 AM after overnight fasting. To account for seasonal variation: a) Sampling was balanced across seasons in both groups (CD: 26 spring, 28 summer, 25 autumn, 23 winter; controls: 25/26/25/24); b) Season-adjusted analysis was performed by assigning samples to ‘high-sunlight’ (May–October) or ‘low-sunlight’ (November–April) periods based on geographic latitude (32°N).

### Determination of CD activity

CD activity was calculated using biochemical and clinical data. Endoscopic scores (SES-CD) and clinical scores (CDAI, HBI) were used to identify four types of disease activity: mildly active (SES-CD 3-6, HBI 5-7), moderately active (SES-CD 7-15, HBI 8-16), highly active (SES-CD > 15, HBI > 16), and remission (SES-CD < 2, HBI ≤ 4). A single physician performed each colonoscopy and assessed the endoscopic scores (26).

### Clinical outcome

Before therapy, fasting blood was drawn between 7:00 and 8:00 in order to evaluate a number of biomarkers. After 20 to 30 minutes in a water bath at 37°C, the blood was centrifuged at 1580g for 5 minutes to collect serum. Using Synchron reagents from Leadmanbio (China), biochemical parameters, including hemoglobin (Hb; g/L), total protein (TP; g/L), albumin (ALB; g/L), high-density lipoprotein cholesterol (HDL-C; mmol/L), and low-density lipoprotein cholesterol (LDL-C; mmol/L), were analyzed using a 7600–210 automatic biochemistry analyzer (Hitachi Inc., Japan). Serum 25(OH)D was measured using radioimmunoassay kits (Beijing North Institute of Biotechnology, China). Standardized methods were used for all biochemical measurements. The definitions of VD deficit, insufficiency, and sufficiency were 25(OH)D <20 ng/mL, 25(OH)D = 20–29 ng/mL, and 25(OH)D ≥30 ng/mL, respectively (27). Patients’ body weight was recorded to the closest 0.1 kg using an electronic scale while they were wearing very little clothes and no shoes. A stadiometer was used to measure height to the closest 0.1 cm, and BMI was computed as weight (kg) divided by height (m). According to WHO guidelines, BMI (kg/m2) values were classified as low (<18.5 kg/m2), eutrophic (18.5-24.9 kg/m2), overweight (25-29.9 kg/m2), or obese (>30 kg/m2) (28, 29). The Harvey-Bradshaw index for CD was the clinical metric employed in this investigation.

### Prognostic nomogram analysis

For multivariate logistic regression, we verified linearity between continuous predictors (age, disease duration) and the log-odds of vitamin D deficiency using the Box-Tidwell procedure (all p > 0.05), confirmed absence of multicollinearity through variance inflation factors (mean VIF = 1.7), and evaluated model fit with the Hosmer-Lemeshow test (p = 0.24). Multivariate data was reduced and risk factors were chosen using the LASSO approach. The variables in the training set were chosen using non-zero LASSO regression coefficients. LASSO regression was performed using R v4.3.1 (glmnet package), with 10-fold cross-validation repeated three times to determine the optimal penalization parameter (λ = 0.032). All continuous variables were standardized (mean = 0, SD = 1) before regularization to ensure coefficient comparability. Selected variables from the LASSO regression model were subjected to multiple logistic regression analysis to construct a prediction model. The non-stick coating nomogram’s calibration was evaluated using a calibration curve, and its discriminatory power was evaluated using the Harrell C index to determine if decision curve analysis is required in order to evaluate net benefit and find clinically relevant non-adherent nomograms. The clinical factor model’s diagnostic effectiveness was evaluated using the matching ROC curve (AUC) from the training and validation toolbox (30).

### Statistical analysis

Data analysis was done using statistical tools. Each variable’s normality was assessed using the Shapiro-Wilk test. Measurements with a normal distribution are displayed as mean ± standard deviation (SD); groups were compared using independent samples t-tests. Groups were compared using non-parametric testing, and non-normal results are displayed as the median (Q1, Q3). Percentages were used to display categorical data. The index with P < 0.1 was added to the list of significant variables for logistic or linear regression analysis after each index completed univariate analysis. P < 0.05 was established as the cutoff point for statistical significance. To investigate the variation in treatment outcomes and influencing factors among various patient categories, subgroup analyses were conducted. The final multivariate model has a significant P value of 0.05.

## Results

### Clinical characteristics

**Figure 1:**
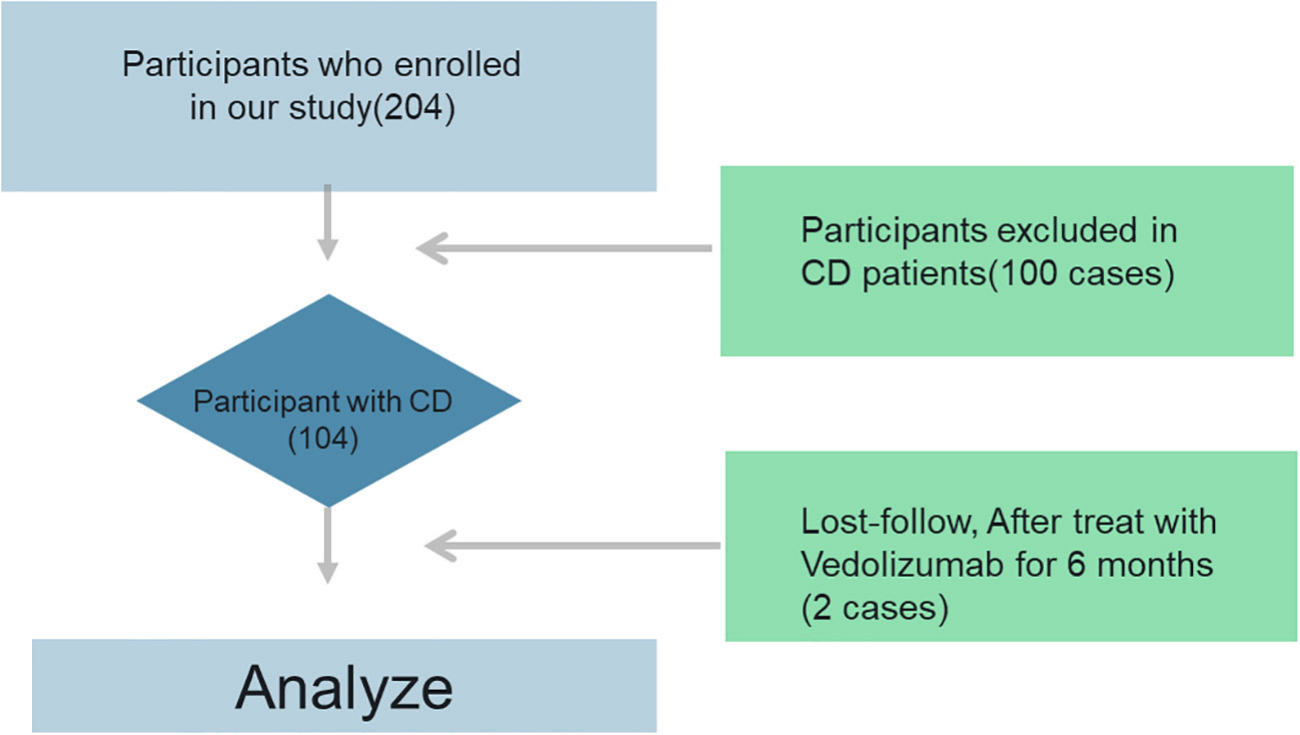
Flowchart of inclusion and exclusion.

Our study comprised 102 patients with CD and 100 healthy volunteers, whose baseline characteristics are displayed in **Table 1**. Fifty-one of the 102 CD patients were in active CD, and fifty-one were in remission. The CD patients were 38.91 ± 3.31 years old, while the healthy volunteers were 38.64 ± 2.26 years old. The two groups’ levels of iron (p = 0.02) and vitamin B12 (p = 0.04) differed significantly. In addition, there were no significant statistical differences in white blood cell count, haemoglobin, platelet count, neutrophils, C-reactive protein, and trace elements (magnesium and ferritin) between the two groups. Lastly, we discovered that individuals with CD had significantly lower vitamin D levels (19.62 ± 2.68) than the healthy control group (22.68 ± 4.61) (**Figure 1**).

**Table 1:** General characteristics of the participants.

Variables	HC (n=100)	CD (n=102)	P value	Active CD (n=51)	CD remission(n=51)	P value
Age (years), mean ± SD	38.64 ± 2.26	38.91 ± 3.31	0.36	38.15 ± 4.62	39.01 ± 2.06	0.21
Duration from disease onset to vitamin D measurement, year,mean ± SD	2.15 ± 0.25	2.09 ± 0.34	0.15	2.08 ± 0.36	2.10 ± 0.14	0.14
Sex, male, n (%)	52	49	0.33	25	24	0.42
Recent steroids, n (%)	&-	22	&-	10	12	0.17
Recent 5-aminosalicylic acid, n (%)	&-	75	&-	40	35	0.28
Recent biologics, n (%)	&-	11	&-	6	5	0.17
Recent azathioprine, n (%)	&-	21	&-	16	5	0.25
Harvey–Bradshaw Index	&-	7.25 ± 1.11	&-	7.82 ± 1.32	3.62 ± 0.95	0.01
Abdominal surgery, n (%)	&-	32	&-	9	23	0.04
CD location, n (%), L1/L2/L3/L4	&-	35/12/50/5	&-	&-	&-	&-
CD behavior, n (%), B1/B2/B2p B3/B3p	&-	30/40/4/17/11	&-	&-	&-	&-

&, no data.

The median number of patients in the CD group had treatment before beginning vedolizumab. The difference in clinical outcomes between individuals with active CD and those in remission was next examined. Significant differences were seen between the CD remission and active CD groups in terms of C-reactive protein (p = 0.04), vitamin D deficiency (p=0.02) and vitamin D sufficiency (p=0.01), and abdominal surgery (p=0.04) (**Table 2**).

**Table 2:** Laboratory data characteristics of the participants.

Variables	HC (n=100)	CD (n=102)	P value	active CD (n=51)	CD remission(n=51)	P value
Laboratory data						
White blood cell count, 103/μL, mean ± SD	7.62 ± 0.65	7.84 ± 0.88	0.62	7.40 ± 0.31	7.36 ± 0.33	0.13
Hemoglobin, g/dL, mean ± SD	13.52 ± 1.40	13.08 ± 0.98	0.44	13.08 ± 0.89	13.11 ± 0.45	0.12
Platelet count, 103/μL, mean ± SD	298.15 ± 85.14	294.15 ± 71.06	0.11	285.64 ± 73.31	290.18 ± 63.25	0.36
Neutrophil, %, mean ± SD	65.28 ± 10.20	64.15 ± 8.62	0.22	63.81 ± 10.32	61.25 ± 9.65	0.09
Lymphocyte, %, mean ± SD	24.68 ± 3.65	22.38 ± 4.78	0.39	24.61 ± 3.33	24.61 ± 2.59	0.12
Albumin, g/dL, mean ± SD	4.62 ± 0.85	4.81 ± 0.75	0.42	4.90 ± 0.62	4.80 ± 0.95	0.32
C-reactive protein, mg/dL, mean ± SD	0.13 ± 0.09	0.18 ± 0.05	0.08	0.19 ± 0.03	0.12 ± 0.06	0.04
Iron (43~172 µg/dL)	79.62 ± 2.61	61.02 ± 2.66	0.02	60.25 ± 0.62	63.25 ± 0.85	0.02
Folic acid(3.1~20.5 ng/mL)	8.69 ± 1.15	8.66 ± 1.62	0.26	8.56 ± 1.25	8.67 ± 0.56	0.09
Vitamin B 12(187~883 pg/mL)	664.86 ± 125.62	658.62 ± 125.02	0.04	602.64 ± 102.62	663.25 ± 58.36	0.15
Zinc(66~ 110 µg/dL)	76.35 ± 6.62	75.62 ± 3.26	0.32	70.02 ± 1.25	76.65 ± 2.61	0.25
Magnesium(Female 19–2.5, Male 1.8–2.6 mg/dL)	2.61 ± 0.36	2.01 ± 0.32	0.15	2.01 ± 0.22	2.12 ± 0.36	0.33
Ferritin(Female: 24~307 ng/mL, Male: 24~336 ng/mL)	77.69 ± 18.62	76.62 ± 21.68	0.08	72.61 ± 6.15	76.35 ± 2.61	0.14
25-OH vitamin D total, ng/mL, mean ± SD	22.68 ± 4.61	19.62 ± 2.68	0.02	18.61 ± 2.68	22.69 ± 2.36	0.01
Level of vitamin D, n (%)						
Deficiency (< 20 ng/mL)	13	24	0.02	18	6	0.02
Insufficiency (20–29 ng/mL)	21	62	0.01	32	30	0.36
Sufficiency (≥ 30 ng/mL)	66	16	0.04	1	15	0.01

### The role of vitamin D levels in CD patients

The similarities between the vitamin D-deficient and normal groups are displayed in **Table 3**. Compared to the group without vitamin D insufficiency, the vitamin D-deficient group had fewer instances of clinical remission (p=0.02) and more males (18 cases) (p=0.01). Regarding clinical medicine usage, the results indicated that the group with vitamin D insufficiency had significantly higher levels of current 5-aminosalicylic acid (p=0.04) and steroids (p=0.02) than the group without vitamin D deficiency. Clinical test results revealed that the vitamin D-deficient group had significantly lower 25-OH vitamin D totals (p=0.01) and significantly higher white blood cell counts (p=0.02) than the non-vitamin D-deficient group. The two groups did not vary substantially in terms of hemoglobin, platelet count, neutrophil count, lymphocyte count, albumin, C-reactive protein, or abdominal surgery.

**Table 3:** Baseline characteristics of the CD population (comparisons between the groups with and without vitamin D deficiency).

Variables	Without deficiency (n = 78)	With deficiency (n = 24)	P value
Age (years), mean ± SD	38.62 ± 2.66	39.26 ± 1.85	0.07
Duration from disease onset to vitamin D measurement, year,mean ± SD	2.03 ± 0.62	2.15 ± 0.55	0.25
Sex, male, n (%)	31	18	0.01
Clinical remission, n (%)	45	6	0.02
Recent steroids, n (%)	18	4	0.02
Recent 5-aminosalicylic acid, n (%)	52	23	0.04
Recent Vedolizumab, n (%)	5	6	0.15
Recent azathioprine, n (%)	15	6	0.11
Laboratory data			
White blood cell count, 103/μL, mean ± SD	7.62 ± 0.62	8.54 ± 0.32	0.02
Hemoglobin, g/dL, mean ± SD	13.69 ± 0.96	13.25 ± 0.25	0.38
Platelet count, 103/μL, mean ± SD	296.34 ± 70.51	296.45 ± 61.32	0.44
Neutrophil, %, mean ± SD	64.32 ± 4.62	64.99 ± 4.36	0.18
Lymphocyte, %, mean ± SD	22.06 ± 0.62	23.61 ± 0.96	0.25
Albumin, g/dL, mean ± SD	4.56 ± 0.62	4.68 ± 0.54	0.31
C-reactive protein, mg/dL, mean ± SD	0.12 ± 0.03	0.13 ± 0.02	0.08
25-OH vitamin D total, ng/mL, mean ± SD	27.61 ± 2.61	15.95 ± 1.62	0.01
Abdominal surgery, n (%)	21	11	0.19

The results before and after using Vedolizumab were then compared in **Table 4**. After six months of vedolizumab treatment for Crohn’s disease, we found statistically significant improvements in the Harvey-Bradshaw index (p=0.01), ESR (p=0.02), white blood cell count (p=0.03), vitamin B12 (p=0.03), magnesium (p=0.03), and vitamin D (p=0.02).

**Table 4:** Crohn’s disease pre- and post-vedolizumab clinical, endoscopic, and laboratory values.

Characteristic	Pre-Vedolizumab(11 case)	Post-Vedolizumab(11 case)	p
Harvey–Bradshaw Index	7.62 ± 1.06	2.03 ± 0.24	0.01
ESR (mm/h)	16.35 ± 2.61	6.02 ± 1.66	0.02
White blood cell count, 103/μL, mean ± SD	7.40 ± 0.31	4.65 ± 0.66	0.03
Hemoglobin, g/dL, mean ± SD	13.08 ± 0.89	13.02 ± 0.77	0.45
Platelet count, 103/μL, mean ± SD	285.64 ± 73.31	286.65 ± 36.15	0.26
Neutrophil, %, mean ± SD	63.81 ± 10.32	60.25 ± 6.32	0.45
Lymphocyte, %, mean ± SD	24.61 ± 3.33	23.65 ± 1.08	0.44
Albumin, g/dL, mean ± SD	4.35 ± 0.62	4.62 ± 1.03	0.32
C-reactive protein, mg/dL, mean ± SD	0.19 ± 0.03	0.26 ± 0.52	0.11
Iron (43~172 µg/dL)	60.25 ± 0.62	60.23 ± 0.15	0.68
Folic acid(3.1~20.5 ng/mL)	8.23 ± 1.25	7.01 ± 0.62	0.55
Vitamin B 12(187~883 pg/mL)	602.64 ± 102.62	501.56 ± 19.25	0.03
Zinc(66~ 110 µg/dL)	70.02 ± 1.25	60.15 ± 2.63	0.15
Magnesium(Female 19–2.5, Male 1.8–2.6 mg/dL)	2.01 ± 0.22	1.11 ± 0.62	0.03
Ferritin(Female: 24~307 ng/mL, Male: 24~336 ng/mL)	72.61 ± 6.15	70.26 ± 2.26	0.15
25-OH vitamin D total, ng/mL, mean ± SD	18.61 ± 2.68	26.62 ± 1.62	0.02

### Univariate, multivariate, and LASSO regression analysis models

**Table 5** lists the risk factors for vitamin D deficiency. Significant differences between patients with and without vitamin D deficiency were found in age (OR: 0.95, 95% CI 0.26-1.33, p=0.01), sex (OR: 0.26, 95% CI 0.25-0.99, p=0.03), newer biologics (OR: 0.54, 95% CI 0.44-1.25, p=0.02), iron (OR: 0.89, 95% CI 0.72-1.62, p=0.02), and total 25-OH vitamin D (OR: 1.25, 95% CI 1.02-1.99, p=0.02). Furthermore, multivariate analysis revealed that after multivariate adjustment, odds ratios were significantly lower for increasing age at diagnosis (OR: 0.12, 95% CI 0.03-0.85, p=0.02), sex (OR: 0.58, 95% CI 0.44-0.95, p=0.01), iron (OR: 0.44, 95% CI 0.11-0.62, p=0.01), and 25-OH vitamin D total (OR: 0.48, 95% CI 0.25-0.95, p=0.03).

**Table 5:** Logistic regression analysis between patien with or without vitamin D deficiency.

Characteristics	Univariate		Multivariate	
	p-Value	OR (95% IC)	p-Value	OR (95% IC)
Age (years), mean ± SD	0.01	0.95 (0.26-1.33)	0.02	0.12 (0.03-0.85)
Duration from disease onset to vitamin D measurement, year,mean ± SD	0.11	0.55 (0.44-1.11)		
Sex, male, n (%)	0.03	0.26 (0.25-0.99)	0.01	0.58 (0.44-0.95)
Clinical remission, n (%)	0.33	0.02 (0.01-0.85)		
Recent steroids, n (%)	0.28	0.99 (0.58-1.62)		
Recent 5-aminosalicylic acid, n (%)	0.32	0.85 (0.25-1.11)		
Recent biologics, n (%)	0.02	0.54 (0.44-1.25)		
Recent azathioprine, n (%)	0.21	1.26 (1.02-1.99)		
Iron (43~172 µg/dL)	0.02	0.89 (0.72-1.62)	0.01	0.44 (0.11-0.62)
Folic acid (3.1~20.5 ng/mL)	0.24	0.45 (0.22-0.95)		
Vitamin B 12 (187~883 pg/mL)	0.15	0.55 (0.42-0.99)		
Neutrophil, %, mean ± SD	0.11	0.24 (0.15-0.65)		
Lymphocyte, %, mean ± SD	0.25	0.62 (0.33-1.98)		
Albumin, g/dL, mean ± SD	0.22	0.32 (0.12-0.85)		
C-reactive protein, mg/dL, mean ± SD	0.65	0.86 (0.71-1.36)		
25-OH vitamin D total, ng/mL, mean ± SD	0.02	1.25 (1.02-1.99)	0.03	0.48 (0.25-0.95)
Abdominal surgery, n (%)	0.09	0.95 (0.36-1.25)		

**Figure 2:**
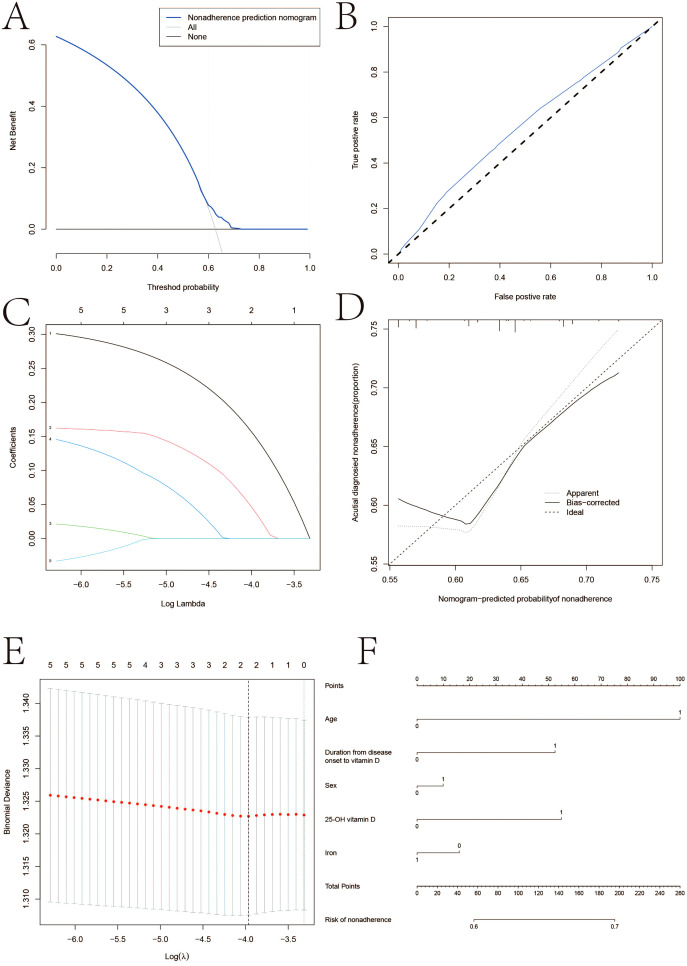
LASSO regression analysis. **(A)** Decision curve analysis for the nonadherence nomogram **(B)** Calibration curves of the nonadherence nomogram prediction in the cohort. **(C)** LASSO coefficient profiles of the 22 characteristics. **(D)** Acutial diagnosied nonadherence. **(E)** Optimal parameter (lambda) selection in the LASSO model uses fivefold cross‐validation using minimal criteria. **(F)** Vertical line was drawn at the value determined using fivefold cross‐validation, where optimum lambda resulted in five features with nonzero coefficients.

**Figure 2** illustrates the correlation between age, sex, iron, and the total amount of 25-OH vitamin D using several linear regression models. Based on CD patients in the vitamin D-positive and vitamin D-negative cohorts, the LASSO regression model reduced 15 components to 5 possible predictors with non-zero coefficients. Age, sex, iron, and total 25-OH vitamin D are some of these traits. The nomogram was created using a model that included the independent predictors previously described. In this cohort, there was significant agreement with the calibration curve of the risk of non-adherence nomogram, which is used to predict risk in patients with CD. The bootstrap validation of 0.912 and the C-index of the cohort-projected nomogram of 0.965 (95% CI, 0.33-2.67) demonstrate the great discriminativeness of the model. With an area under the curve (AUC) of 0.905, the CD linkage indicator nomogram’s decision curve analysis is displayed. In summary, 25-OH vitamin D levels, age, and the time between the development of the illness and vitamin D insufficiency may be helpful indicators for CD patients.

## Discussion

Crohn’s disease (CD), a recurrent inflammatory bowel disease (IBD), is caused by innate immune dysregulation in the gut (31). Patients with CD may be at higher risk of developing the disease if they have a vitamin D deficit and the hazards that come with it. (32). According to our findings, vitamin D levels in CD patients significantly decreased. Among individuals with vitamin D insufficiency, there were statistically significant variations in white blood cell count, clinical remission, and gender. Following vedolizumab therapy, vitamin D levels considerably increased. In univariate, multivariate, and LASSO regression models, common characteristics, including age and vitamin D levels, may be risk factors for the advancement of CD illness.

With 20% coming from food, vitamin D is a steroid hormone with immunomodulatory properties that is mostly produced in the skin following sun exposure (33, 34). Prior research has demonstrated a substantial correlation between vitamin D insufficiency and the prevalence of CD in patients (32). Vitamin D production may be negatively impacted by darker-skinned Asian individuals’ decreased sun penetration (35, 36). Vertebral fractures are more frequent in CD patients than in healthy people, and they are crucial for children’s bone development (37). Prior to beginning steroid therapy, it is critical to ascertain the severity of vitamin D deficiency (38). While pan-hypovitaminosis occurred, vitamin D’s unique link to inflammation suggests its role as both a biomarker and pathophysiological mediator. For CD patients with active illness or undergoing steroid treatment, vitamin supplementation and monitoring are advised (38). It is still unknown, nevertheless, how precisely vitamin D levels relate to clinical remission or activity in CD patients.

Among our CD group, vedolizumab medication levels were significantly greater among those with higher vitamin D levels. From a therapeutic perspective, raising vitamin D levels might be one method of raising medication levels. However, due to enhanced absorption, mucosal healing, and general improvements in illness and nutritional condition, vedolizumab could have indirectly increased vitamin D levels (39). Compared to the low pre-treatment vedolizumab group, the vitamin D levels of the pre-treatment vedolizumab patients are lower (40). While our findings elucidate vitamin D pathophysiology in CD—particularly relevant given its ileal predominance—similar mechanisms may operate in UC through distinct pathways (e.g., epithelial barrier repair). Future studies should validate whether the identified risk factors (age, disease duration, baseline vitamin D) generalize across IBD subtypes.

Circulating 25D levels were determined using lifestyle surveys and direct measurements of plasma 25D, according to a large prospective cohort research of 72,719 women who participated in the Nurses’ Health Study, which was published in 2011 (41). Between 1986 and 2008, the ladies were monitored, and 122 CD cases were noted throughout that period. A combination of lifestyle surveys and direct measurements of plasma 25D was used to evaluate the levels of circulating 25D. The authors draw the conclusion that in this female group, a higher projected amount of circulating 25D considerably lowers the incidence of CD. Similarly, a retrospective cohort study of 403 patients with CD concluded that vitamin D deficiency is common in the patient population and is independently associated with lower health-related quality of life (HRQOL) and higher disease activity (42). Similarly, a retrospective cohort analysis of 403 CD patients found that vitamin D deficiency is common in the patient population and is independently associated with lower health-related quality of life (HRQOL) and greater disease activity (43). It should be highlighted, nonetheless, that in this particular demographic, vitamin D insufficiency may result from malabsorption and inactivity brought on by active illness before the clinical presentation (44). When vitamin D levels and indicators of inflammation and disease activity were analyzed in CD patients, fecal calprotectin assays revealed a substantial negative connection between 25D concentrations and intestinal inflammation (45). Patients in clinical remission are affected by this connection, whereas those with active CD are not. Moreover, the 25D level was not associated with either disease activity ratings or systemic inflammation as assessed by circulating C-reactive protein in this cohort (46). Our findings indicate that low vitamin levels are significantly associated with CD and active CD.

Age, gender, and vitamin D levels were the main risk factors for CD patients in our study. Previous studies have shown a considerable decrease in vitamin D levels in women. Han et al. found that vitamin D insufficiency was associated with Crohn’s disease (p=0.012) and female sex (p=0.012) (47). Women were 1.7 times more likely than men to have a 25-OH vitamin D level below 20 ng/ml, per Samantha et al.’s research (48). Women are a risk factor, according to our findings. Other investigations, however, have not demonstrated this correlation (49, 50). Vitamin D insufficiency is linked to longer age at diagnosis, according to research by Alex Ulitsky et al (42). According to our research, one risk factor for vitamin D insufficiency is being young when diagnosed. Furthermore, it has been demonstrated that a younger age of onset is a poor prognostic factor for IBD and will need more future medical care (26, 51). Our results point to the necessity of screening our younger population for vitamin D (52, 53). Vitamin D supplementation is useful in raising vitamin D concentrations and lowering CRP levels in adults (54) and pediatric IBD patients (55). On the other hand, 82% of IBD patients had untreated vitamin D insufficiency, indicating a clinical disregard for the issue (56). While pan-hypovitaminosis occurred, vitamin D’s unique link to inflammation suggests its role as both biomarker and pathophysiological mediator (57, 58). While vedolizumab’s gut-specific action provides a unique model for studying mucosal healing-nutrient interactions, our results conceptually align with evidence for anti-TNF therapies. Future studies should directly compare vitamin D trajectories across biologic classes.

Furthermore, we discovered that vitamin D and iron ion levels can possibly be significant risk factors for CD patients. Moreover, disease activity is linked to vitamin D insufficiency. According to Søren et al., the median 25-OH vitamin D levels in slightly, moderately, and remission-stage Crohn’s disease were 64, 49, and 21 nmol/l (p<0.01) and 68, 76, and 35 nmol/l (p<0.05) by the Crohn’s disease activity index, respectively. Furthermore, low serum 25-OH vitamin D was linked to active Crohn’s disease. Regardless of disease activity, individuals who smoked had lower 25-OH vitamin D levels than nonsmokers (59). Vitamin D replenishment is linked to lower disease activity and higher quality of life in CD patients, according to Samantha et al. (48). A meta-analysis of 27 studies, including 8316 IBD patients, by Gubatan et al. found a significant correlation between low vitamin D levels and disease activity (OR: 1.53, 95% CI 1.32-1.77) (60). In addition to clinical evaluation, studies have demonstrated a negative correlation between vitamin D levels and inflammatory markers in CD populations, such as white blood cell count, CRP, and fecal calprotectin (61–64). Although standardized low-dose supplementation introduces uniformity, unmeasured individual factors (e.g., adherence, baseline deficiency severity) may contribute residual variability. The effect size and mechanistic plausibility support vedolizumab’s role, but prospective trials with controlled supplementation are warranted.

### Limitations

The very limited number of instances in each group we examined is the first of many limitations of our research that should be addressed. Firstly, we mitigated recall bias via standardized electronic health record abstraction and sensitivity analyses excluding inconsistently documented cases. Secondly, the small size of our studies does not provide sufficient evidence to support clinical outcomes, and further expansion of the sample size for analysis data is needed in future studies. Residual effects of diet/sun exposure remain possible despite seasonal adjustment. Thirdly, we did not examine the effects of vitamin D supplementation in individuals with CD since our research was a retrospective observational study. Despite this, our research focuses on the connection between CD illness and vitamin D levels in local populations. But there is also a need to consider the effects of factors such as sunlight on vitamin D in future studies. Lastly, we examine the connection between CD and a few key clinical nutrients; nevertheless, we still need to investigate the association between vitamin D and nutrients.

## Conclusion

According to this study, vitamin D insufficiency was often linked to CD patients with active status and pre-treatment Vedolizumab. Furthermore, Age, time since illness onset, and 25-OH vitamin D were found to be significant risk factors for CD. Our research serves as a reference for the connection between CD and vitamin D insufficiency on the Chinese mainland and might guide the clinical diagnosis and management of vitamin D-deficient CD patients.

## Data Availability

The original contributions presented in the study are included in the article/supplementary material. Further inquiries can be directed to the corresponding author.
